# Comparative Study of the Effectiveness of Cellulose, Pectin and Citrus Peel Powder in Alleviating Loperamide-Induced Constipation

**DOI:** 10.3390/foods15020240

**Published:** 2026-01-09

**Authors:** Feiyang Yang, Ge Wang, Miner Huang, Xin Liu, Sheng Tang, Wenjuan Li, Yuanli Luo, Junying Bai, Linhua Huang

**Affiliations:** 1 Citrus Research Institute, Southwest University, Chongqing 400700, China; 18602383387@163.com (F.Y.); wangge0113@163.com (G.W.); 13829134781@163.com (M.H.); lxqq1985734352@email.swu.edu.cn (X.L.); t2353749207@163.com (S.T.); 2 National Citrus Engineering Research Center, Chongqing 400700, China; 3 State Key Laboratory of Food Science and Resources, Nanchang University, Nanchang 330047, China; wenjuanli@ncu.edu.cn; 4 Southeast Chongqing Academy of Agricultural Sciences, Chongqing 408000, China; luoyuanli163@163.com

**Keywords:** citrus, dietary fiber, pectin, solubility, functional food

## Abstract

Constipation is a global health issue, with a prevalence of approximately 16%, and insufficient dietary fiber intake is a major contributing factor. Citrus peel residue contains a high proportion of dietary fiber, accounting for about 20–44% of its composition. In this study, the constipation-relieving effects of three functional components derived from citrus peel residue—cellulose (CEL), pectin (PEC), and citrus peel powder (CPP)—were systematically compared using a loperamide-induced mouse model. All groups were administered an equivalent dose of 200 mg/kg daily. The results showed that supplementation with CEL, PEC, and CPP improved defecation parameters. Among these, PEC effectively modulated the SCF/C-kit and Nrf2/HO-1 pathways. Compared with the model group, PEC increased *Akkermansia* abundance by approximately 34% and reduced *Desulfovibrio* abundance by about 26% Additionally, the smaller particle size and improved solubility of PEC promote the production of beneficial metabolites, thereby alleviating constipation. In contrast, CEL primarily alleviates constipation through its physical properties. At equivalent doses, CPP provides less constipation relief due to its lower component concentrations and a primary composition of insoluble dietary fiber. These findings provide preliminary mechanistic insights and support further exploration of citrus by-products as functional food candidates for the management of constipation.

## 1. Introduction

Constipation is a common gastrointestinal disorder characterized by difficulty defecating, decreased bowel movement frequency, and hard, dry stools. It is estimated that constipation affects approximately 9.5% of adolescents worldwide, with a global prevalence ranging from 11% to 20%. It is more prevalent among adults, affecting around 16% of the population [1]. Commonly recommended preventive measures include dietary modification, increased physical activity, and establishing regular bowel habits. Chronic constipation may lead to gut microbiota dysbiosis and may even contribute to cardiovascular diseases such as hypertension and stroke [2]. Judkins et al. found a positive correlation between constipation and cardiovascular diseases such as stroke. Patients with constipation, particularly the elderly, experience sustained increases in blood pressure during bowel movements [3]. When constipation occurs, individuals frequently resort to treatments such as laxatives and intestinal secretagogues [4]. However, long-term use of these medications can cause adverse effects, including diarrhea, nausea, and abdominal pain, and may ultimately induce intestinal dysfunction or drug dependence, resulting in irreversible harm [5]. Therefore, identifying safe and practical approaches for the prevention and treatment of constipation is essential. The pathogenesis of constipation is multifaceted, and research indicates that it is closely associated with the apoptosis of interstitial cells of Cajal (ICCs). These cells are considered neuromuscular mediators and pacemaker cells that regulate gastrointestinal motility [6]. Their absence results in markedly reduced colonic motility, thereby triggering constipation [7]. It is also notable that the proliferation, differentiation, and function of interstitial cells are closely linked to the activation of the c-Kit receptor tyrosine kinase. This kinase binds to the stem cell factor (SCF) ligand and regulates gastrointestinal motility [8]. Consequently, the number of interstitial cells correlates with the expression levels of SCF and c-Kit within the SCF/c-Kit pathway [6]. Constipation also decreases the activity of antioxidant enzymes such as superoxide dismutase (SOD) and glutathione peroxidase (GPX), thereby elevating oxidative stress, which may promote ICC apoptosis and impair gastrointestinal motility [2]. In addition, increased intestinal inflammation and damage to tight junction proteins disrupt intestinal barrier integrity, further contributing to constipation [9]. Abnormal secretion of intestinal neurotransmitters, including motilin (MTL) and gastrin (GAS), can also precipitate constipation. In recent years, increasing attention has been given to the role of the gut microbiota and its metabolites in the development of constipation. Constipation disturbs microbial homeostasis, characterized by increased levels of harmful bacteria (e.g., *Desulfovibrio*) and reduced levels of beneficial bacteria (e.g., *Lactobacillus*, *Akkermansia*, and *Dubosiella*), alongside an elevated Firmicutes/Bacteroidetes (F/B) ratio [10]. For instance, Yang et al. discovered that *Akkermansia* levels in constipated mice decreased by around 20% [11]. Collectively, these findings indicate that constipation is a complex condition involving the enteric nervous system, the intestinal barrier, the gut microbiota, and associated metabolites.

Citrus fruits are the world’s most widely cultivated fruit and undergo extensive processing. However, this large-scale processing generates substantial quantities of inedible citrus peel, resulting in severe environmental pollution and resource waste [12]. Discarded citrus peel is rich in dietary fiber, which can be classified by solubility as soluble dietary fiber (SDF), including mainly pectin (PEC), and insoluble dietary fiber (IDF), including primarily cellulose (CEL) [13]. Research indicated that PEC, CEL, soluble sugars, and hemicellulose collectively constituted 45% of citrus peel residue by weight [14], with PEC and CEL being the predominant dietary fibers [15]. Dietary fiber supplementation is widely recognized as a key strategy for preventing and alleviating constipation. Studies demonstrate that PEC can downregulate the expression of pro-inflammatory factors, enhance intestinal barrier function, and optimize the gut microbiota and metabolites simultaneously, thereby strengthening intestinal barrier integrity [16,17,18]. Although CEL is structurally dense and challenging to digest and utilize, it increases stool bulk, thereby promoting intestinal motility [19,20]. Previous experiments have shown that, due to its rich flavonoid and fiber content, citrus peel powder (CPP) can effectively prevent and treat non-alcoholic fatty liver disease and enhance intestinal barrier function, making it a promising dietary fiber supplement [21,22]. However, it remains unclear whether CPP can alleviate constipation.

This study used citrus peel as the raw material for the extraction and purification of CEL and PEC. The composition, structure, and functional properties of the CPP, the CEL, and the PEC were characterized. A mouse constipation model was established using loperamide hydrochloride (LOP) to systematically investigate the effects of CEL, PEC, and CPP on constipation relief. The study evaluated their impact on intestinal barrier function and aquaporin expression. Building upon this, the study further compared the differential expression of key genes and proteins in the SCF/C-Kit signaling pathway and the Nrf2/HO-1 antioxidant pathway among the three substances. In combination with an analysis of ICC apoptosis, the study systematically compared the efficacy of constipation relief among the three substances. These differences in efficacy may stem from the composition, structure, and functional characteristics of CEL, PEC, and CPP. Soluble, fermentable PEC may alleviate constipation by activating relevant pathways through modulation of the gut microbiota and increasing beneficial metabolites. Insoluble CEL likely relies primarily on its physical water-holding capacity and swelling properties. The effects of the mixed component, CPP, may stem from the contributions and interactions of its various components. The research objectives and hypotheses contribute valuable insights for the comprehensive utilization of citrus resources and the development of related dietary supplements.

## 2. Materials and Methods

### 2.1. Main Materials

The citrus variety used in this study is Hongyun Xianggan, a novel hybrid citrus cultivar developed by the Citrus Research Institute of Southwest University (Chongqing, China). The citrus peel utilized in the experiments originated from processing residues of Hongyun Xianggan. The total PEC content kit, PEC methylesterification degree kit, CEL content kit, hemicellulose content kit, and lignin content kit were all purchased from Suzhou Greeth Biotech Co., Ltd. (Suzhou, China). Lop was purchased from Shanghai McLean Biotechnology Co., Ltd. (Shanghai, China). All the chemicals and reagents were of analytical grade.

### 2.2. Sample Preparation

Fresh citrus peels were thoroughly washed, cut into small cubes, and dried in an oven at 60 °C for 24 h. The dried material was ground to a fine powder using a pulveriser and stored at −4 °C for subsequent use (Figure S1).

#### 2.2.1. Preparation of PEC

Mix citrus peel powder with water at a solid-to-liquid ratio of 1:10 (g/mL). Then, subject the mixture to ultrasonic treatment at 150 W for 40 min. Centrifuge at 3300× *g* for 15 min at room temperature to collect the precipitate, thereby completely removing the sugar. The precipitate was mixed with a freshly prepared 50% ethanol solution (prepared by mixing analytical-grade anhydrous ethanol and deionized water in a 1:1 volume ratio) at a solid-to-liquid ratio of 1:25 (g/mL). The mixture was sonicated at 150 W for 40 min [23]. Pigments and flavonoids were then removed by centrifugation. The treated residue was then mixed with pure water at a solid-to-liquid ratio of 1:25 (g/mL). Subsequently, 1.5% composite enzyme (α-amylase: neutral protease = 1:3) was added. Incubate at 60 °C for one hour, then inactivate the enzymes by boiling for ten minutes. Mix the treated orange peel residue with a citric acid solution (pH 2.5) and adjust the pH to 2.5. Sonicate at 70 °C for 40 min. The mixture was vacuum filtered while still hot. Once cooled to 40 °C, an equal volume of 95% ethanol was added to precipitate the PEC [24]. After settling overnight, Centrifuge at 3300× *g* for 15 min at room temperature to collect the precipitate. Repeat the water-soluble ethanol precipitation process three times, wash twice with anhydrous ethanol, and then vacuum-dry at 60 °C to obtain the PEC sample. The sample extraction rate was 25.36%, with galacturonic acid content at 74.43% and an esterification degree of 19.23%.

#### 2.2.2. Preparation of CEL

The hot, vacuum-filtered residue was mixed with a 10% sodium hydroxide solution at a solid-to-liquid ratio of 1:15 (g/mL). After ultrasonication for 1.5 h, the mixture was vacuum-filtered, and the residue was washed to neutrality. The mixture was then combined with 10% hydrogen peroxide, and the pH was adjusted to 11 using sodium hydroxide. The mixture was sonicated for a further hour. This process was repeated two to three times until the residue turned white [25,26]. The bleached residue was then washed with pure water and dried at 60 °C, yielding high-purity CEL with an extraction rate of 24% and a purity of 80.6%.

### 2.3. Component Analysis

#### 2.3.1. Basic Component Analysis

Moisture content was determined using the AOAC official method (AOAC 925.09), and ash content was measured according to AOAC 920.138. Protein content was determined by the Kjeldahl method [27], and fat content by Soxhlet extraction [28]. PEC, CEL, hemicellulose, and lignin contents were all measured using specific reagent kits. Specifically, PEC was expressed as galacturonic acid, which was determined using the Cazol Colorimetric Method. The degree of methyl esterification was represented by the ratio of methanol yield to galacturonic acid content. CEL content was determined using the potassium dichromate-ferrous ammonium sulphate method. Hemicellulose content was determined by combining hydrochloric acid hydrolysis with the DNS sugar determination method. Lignin was determined using the concentrated sulfuric acid method. The content of galacturonic acid in CPP was determined by the Cazol Colorimetric Method. The SDF-to-IDF ratio in CPP was determined using the method of Yang et al. [29], with certain modifications. In brief, CPP was incubated in water at 90 °C for 1 h, then stirred at room temperature for 2 h. The supernatant was separated by centrifugation. The precipitate was dried and weighed to obtain IDF. The supernatant was mixed with fourfold the volume of 95% ethanol, precipitated for 12 h, and the precipitate was collected, freeze-dried, and weighed to obtain SDF. The corresponding results were obtained by calculating the SDF-to-IDF ratio.

#### 2.3.2. Determination of Total Phenolic and Total Flavonoid Content

The total phenolic content was measured using the Folin–Ciocalteu method and expressed as a gallic acid equivalent (mg/g). The results were averaged from three replicates [30]. Total flavonoid content was determined using the aluminum nitrate colorimetric method, with the results expressed as rutin concentration (mg/mL). Three measurements were taken and the average value calculated [20].

#### 2.3.3. Determination of Flavonoid Compounds

Flavonoid determination was performed using the Mesquita method with minor modifications [31]. Specifically, 20 µL of the sample was injected into an Eclipse XDB-C18 column (Agilent Technologies, Santa Clara, USA) (4.6 × 250 mm) on an Ultimate 3000 system (Thermo Fisher Scientific, Bremen, Germany), with detection at 283 nm. The column temperature was maintained at 30 °C, and the flow rate was 1 mL/min. The following gradient elution program was employed: 0–25 min: 82% B; 20–30 min: 82–68% B; 30–40 min: 68–55% B; 40–48 min: 55–32% B; 48–58 min: 32–0% B; 58–68 min: 0–82% B. The mobile phase consisted of acetonitrile (B) and 1% acetic acid (A). All solvents were HPLC grade. Qualitative analysis was performed by comparing the retention times of the detected peaks in the samples with those of the standards. Quantitative analysis of the flavonoid components in the samples was conducted using the external standard method.

### 2.4. Structural Characteristics

#### 2.4.1. Scanning Electron Microscopy Analysis (SEM)

After metal spraying on CEL, PEC, and CPP, their spatial structures were observed at magnifications of 10,000× and 1000× [11].

#### 2.4.2. Fourier Transform Infrared Spectroscopy (FTIR)

FTIR analysis was performed on CEL, PEC, and CPP using a modified FTIR spectrometer [32]. Specifically, a mixture of CEL, PEC, CPP, and potassium bromide is ground and pressed into transparent, thin sheets. These sheets are then scanned 32 times at a resolution of 4.00 cm^−1^ over the wave-number range 500–4000 cm^−1^ [20].

#### 2.4.3. Thermogravimetric Analysis

Thermogravimetric analysis was performed using a Shimadzu Corporation (Japan) DTG-60 series simultaneous TG/DTA system under a nitrogen atmosphere at a heating rate of 10 °C/min over the temperature range of 30–600 °C [33].

#### 2.4.4. Zeta Potential and Particle Size Analysis

Following the method of Shang [34], the sample was diluted to a 0.025% concentration at 25 °C. The zeta potential and particle size were then determined using a Malvern laser particle size analyser (Malvern Instruments, Malvern, UK).

### 2.5. Physicochemical Properties Analysis

#### 2.5.1. Water-Holding Capacity (WHC), Water Solubility Index (WSI), Oil-Holding Capacity (OHC), and Swelling Capacity (SC)

WHC and WSI were determined using methods previously reported with minor modifications [35,36]. A mixture containing 0.5 g each of CEL, PEC, and CPP was dissolved in 25 mL of distilled water and stirred for one hour. The mixture was then centrifuged at 1650× *g* for 10 min at room temperature to remove the supernatant. Dry the supernatant to a constant weight and record the weight as M1. Weigh the remaining precipitate and record the result as M2. The formulas for WHC and WSI are: WHC (g/g) = (M2 − M)/M WSI (g/g) = M1/M.

OHC was determined according to a previously published method with modifications [37]. Mix 0.5 g of CEL, PEC, and CPP (M) with 25 mL of sunflower seed oil for one hour. Centrifuge at 2570× *g* for 10 min at room temperature to remove the supernatant. Absorb the excess oil from the interior of the tube using filter paper, then weigh the precipitate as M1. The OHC formula is: OHC (g/g) = (M1 − M)/M.

SC was determined according to Wang [38] with modifications. Place 0.3 g each of CEL, PEC, and CPP (M) into a graduated cylinder and record the initial volume (V1). Add 5 mL of distilled water to each sample, mix for 24 h, and then measure the final volume (V2). The SC formula is: SC (v/g) = (V2 − V1)/M.

#### 2.5.2. Cholesterol Binding Capacity (CBC)

Slightly modified based on Zhang’s method [39]. Place 1 g each of CEL, PEC, and CPP into a 100 mL volumetric flask. Add 25 g of egg yolk emulsion and mix thoroughly. Adjust the pH to 7, then shake in a water bath at 37 °C for three hours. Centrifuge at 1650× *g* for 20 min at room temperature. Transfer 0.02 mL of the resulting supernatant to a cholesterol standard curve prepared using the o-phthalaldehyde method to determine the concentration of the experimental group sample, C1. The concentration of the untreated egg yolk solution processed by the same method is denoted as C_2_. The concentration of the untreated egg yolk emulsion is denoted as C3. The CBC is calculated as follows: 25{(C2 − C1) − (C3 − C2)}/M.

#### 2.5.3. Total Antioxidant Capacity (ABST and FRAP Methods)

The total antioxidant capacity (ABST and FRAP methods) of CEL, PEC, and CPP was determined using the Total Antioxidant Capacity Test Kit (ABTS method) (Jiancheng Bioengineering Institute, Nanjing, China) and the Total Antioxidant Capacity Test Kit (FRAP method) (Jiancheng Bioengineering Institute, Nanjing, China).

### 2.6. Constipation Animal Experiment Design

All animal experiments were approved by the Laboratory Animal Ethics Committee of Southwest University (protocol code SWU No. IACUC-20251106-02). Thirty-six six-week-old male C57BL/6 mice were obtained from Chongqing Ensiwei Biotechnology Co., Ltd. (Chongqing, China). The mice were housed in animal facilities under SPF conditions, with free access to food and water. All mouse feed was purchased from Chongqing Tengxin Biotechnology Co., Ltd. (Chongqing, China). The feed had an energy ratio of 28.9% protein, 11.9% fat, and 59.2% carbohydrates, with a maximum crude fiber content of 50 g/kg. The temperature was maintained at 22 ± 2 °C under a 12-h light/dark cycle (lights on from 08:00 to 20:00, lights off from 20:00 to 08:00 the following day). Before the experiment, the mice underwent a 1-week acclimatization period. After the acclimatization period, all mice were stratified by body weight and randomly assigned to six groups (*n* = 6) using a random assignment method: a control (CT) group; a Lop model (MD) group (10 mg/kg) [40,41,42], CEL group (200 mg/kg), PEC group (200 mg/kg) [43,44], CPP group (200 mg/kg), and positive drug control (PC) polyethylene glycol 4000 powder group (3 g/kg) [5]. From day 7 to day 38, all groups except the CT group received daily oral administration of 10 mg/kg Lop, while the CT group received an equal volume of saline. Mouse body weight and food intake were recorded weekly. Daily observation of mouse status revealed no deaths or significant toxic reactions. In mice outside the CT group, reduced fecal water content and particle count were observed. When the MD group mice exhibited short, hard, and dry feces, the constipation model was successfully established. Following modeling, fresh fecal pellets were collected from each mouse using sterile instruments and rapidly placed in liquid nitrogen for flash-freezing. They were then transferred to a −80 °C freezer for storage, enabling subsequent independent analysis. To avoid assessment bias, researchers remained blind to group information throughout the entire process: from sample collection to subsequent outcome measurement and data analysis.

### 2.7. Defecation and Gastrointestinal Transit Experiments

Defecation experiments in mice were conducted according to previously reported methods, with modifications [45]. On day 38, the mice were fasted for 12 h but had free access to water [42]. On day 39, after the respective treatments had been administered to each group, the mice were transferred to clean, empty individual cages and provided with food and water. Thirty minutes later, all mice received a gavage of an activated charcoal powder solution (0.2 mL). The following parameters were measured: the time (in minutes) from charcoal administration to the first instance of defecation; the time (in minutes) from charcoal administration to the first instance of black stool; and the number and weight of feces excreted per mouse within three hours. Feces from each mouse were collected in sterile polyethylene tubes. The sterile polyethylene tubes were placed in an oven at 55 °C and dried for 24 h. After thorough drying, the moisture content was measured [5,40,41]. On Day 41, after all the mice had fasted for 12 h, each group received the corresponding treatment. Subsequently, all mice were administered activated charcoal powder orally. Twenty-five minutes after the oral administration of the activated charcoal powder solution, the mice were euthanized and dissected to obtain their small intestines. The total length of the small intestine and the distance traveled by the activated charcoal powder were measured [40,46].

### 2.8. Histopathological Staining

This was slightly modified according to Liu’s method [47]. Specifically, colon sections were subjected to haematoxylin and eosin (H&E) staining and alizarin red-periodic acid-Schiff (AB-PAS) staining (*n* = 4). The sections were then observed at room temperature under an inverted light microscope (Zeiss Axio Vert A1, Carl Zeiss, Oberkochen, Germany). Determining crypt depth and goblet cell count using ImageJ (ImageJ 1.54p).

### 2.9. Immunofluorescence Staining

The determination was performed according to previously published methods [48]. The paraffin sections were dewaxed and then subjected to antigen retrieval with an antigen retrieval solution (*n* = 3). The tissue sections were blocked with 3% BSA for 30 min, then incubated overnight at 4 °C with the c-kit primary antibody. After washing with PBS, the sections were incubated with the secondary antibody at room temperature in the dark for 50 min. To detect cell death in ICCs, a 1-h incubation with the TUNEL assay kit was performed at 37 °C. The nuclei were then counterstained with DAPI, and the slides were sealed with an antifluorescence quenching mounting medium. Observations were performed at room temperature using an inverted light microscope (Zeiss Axio Vert A1, Carl Zeiss, Oberkochen, Germany). Analyze its fluorescence intensity using ImageJ (ImageJ 1.54p).

### 2.10. RNA Extraction and Real-Time Quantitative PCR Analysis (qRT-PCR)

RNA was isolated from colon tissue using Trizol reagent. These samples were reverse transcribed into cDNA using a reverse transcription kit (Biogen Japan Co., Ltd., Tokyo, Japan) [47]. Reaction mixtures were prepared using SYBR Green real-time quantitative PCR reagents (Takayasu Bioengineering Co., Ltd., Tokyo, Japan), with β-actin as the internal control. The relative expression levels of the internal control and target genes in colon tissue were then calculated using the 2^−ΔΔCT^ method. Detailed RT-PCR primer sequences are provided in Table S2 below.

### 2.11. Western Blot

GAPDH antibodies were purchased from MCE (Monmouth Junction, NJ, USA). Antibodies against c-Kit, Nrf2, and HO-1 were purchased from Cell Signalling Technology (Danvers, MA, USA). Western blotting was performed as previously described [47]. The protein bands were normalized against GAPDH as a reference.

### 2.12. Gut Microbiome Analysis

Extract metagenomic DNA from fecal samples using a rapid DNA centrifugation kit (MJYH, Shanghai, China) (*n* = 6). Subsequently, amplify the V3–V4 region of the 16S rDNA gene via PCR (primers: 338F: 5′-ACTCCTACGGGAGGCAGCAG-3′, 806R: 5′-GGACTACHVGGGTWTCTAAT-3′). Amplification conditions were as follows: 3 min pre-denaturation at 95 °C, 30 cycles (denaturation at 95 °C for 30 sec, annealing at 55 °C for 30 s, extension at 72 °C for 45 s), followed by a 10-min extension at 72 °C, and finally stored at 4 °C (PCR instrument: ABI GeneAmp^®^ 9700, Applied Biosystems, Foster City, CA, USA). The PCR reaction mix consisted of: 4 μL 5× TransStart FastPfu Buffer, 2.5 mM dNTPs (2 μL), upstream primer (5 μM) (0.8 μL), downstream primer (5 μM) (0.8 μL), TransStart FastPfu DNA Polymerase (0.4 μL), template DNA (10 ng), and made up to 20 μL. Products were purified using a DNA gel extraction kit (MJYH, Shanghai, China). Post-assembly-optimised sequences were denoised using the DADA2 plugin in the QIIME 2 workflow with default parameters. Sequences processed by DADA2 are typically referred to as ASVs (Amplified Sequence Variants). To minimize the impact of sequencing depth on subsequent α-diversity and β-diversity analyzes, the number of sequences per sample was normalized to 37,770. After normalization, the average sequence coverage (Good’s coverage) per sample remained at 99.09%. Species-level taxonomic analysis of ASVs was performed using the Naïve Bayes classifier in Qiime2, based on the Silva 16S rRNA gene database (v.138.2). All data analyses were conducted on the Meiji Bio Cloud Platform (https://cloud.majorbio.com; accessed on 15 September 2025). Alpha diversity indices, including Sobs and Shannon, were calculated using mothur software (version v.1.30.2) (http://www.mothur.org/wiki/Calculators; accessed on 20 September 2025), and Wilcoxon rank-sum tests were performed to analyse intergroup differences in alpha diversity. Principal coordinate analysis (PCA) based on the Bray–Curtis distance algorithm was employed to assess the similarity of microbial community structures among samples.

### 2.13. Fecal Metabolites Analysis

Fecal samples were placed in centrifuge tubes containing grinding beads (*n* = 6), followed by the addition of an 80% methanol extraction solution containing 0.02 mg/mL internal standard (L-2-chlorophenylalanine). After thorough grinding, the supernatant was transferred to injection vials with inserts for instrument analysis. Chromatographic separation was performed using an UltiMate 3000 UPLC system (Thermo Fisher Scientific, Bremen, Germany). Metabolites eluted from the column were detected using a high-resolution tandem mass spectrometer (Q-Exactive; Thermo Fisher Scientific, Waltham, MA, USA). Chromatographic conditions were as follows: flow rate 0.4 mL/min, column temperature 40 °C, mobile phase A (95% water + 5% acetonitrile, containing 0.1% formic acid), and mobile phase B (47.5% acetonitrile + 47.5% isopropanol + 5% water, containing 0.1% formic acid). After completing the instrument operation, the LC-MS raw data were imported into the metabolomics processing software Progenesis QI (ProgenesisQI v3.0) (Waters Corporation, Milford, CT, USA) for baseline filtering, peak identification, integration, retention time correction, and peak alignment. Yielding a final data matrix containing retention times, mass-to-charge ratios, and peak intensities. Concurrently, MS and MS/MS mass spectrometry data were matched against public metabolite databases HMDB (http://www.hmdb.ca; accessed on 5 November 2025) and Metlin (https://metlin.scripps.edu; accessed on 5 November 2025), as well as MajorBio’s proprietary library, to derive metabolite identifications. The data matrix obtained from the search is uploaded to the MajorBio Cloud platform (https://cloud.majorbio.com; accessed on 11 November 2025) for analysis. Preprocessing of the data matrix is performed as follows: Missing values are removed using the 80% rule, retaining variables with non-zero values in at least 80% of samples. Missing values are then imputed (using the minimum value in the original matrix). To minimize errors arising from sample preparation and instrument instability, the response intensities of sample mass peaks are normalized using sum normalization, yielding a normalized data matrix. Simultaneously, variables with QC sample relative standard deviation (RSD) > 30% were excluded. Log10 transformation was applied to the matrix, yielding the final data matrix for subsequent analysis.

### 2.14. Statistical Analysis

The results are expressed as the mean ± standard error of the mean (SEM). Statistical analysis was performed using GraphPad Prism 8.0 software. Statistical significance was determined using one-way ANOVA with Tukey’s post hoc test for multigroup comparisons and unpaired Student’s *t*-test for two-group analyses. Spearman’s rank correlation was performed using Origin 2021. In this study, *p* < 0.05 was considered statistically significant.

## 3. Results and Discussion

### 3.1. Study on the Composition, Structure, and Functional Properties of CEL, PEC, and CPP

**Figure 1:**
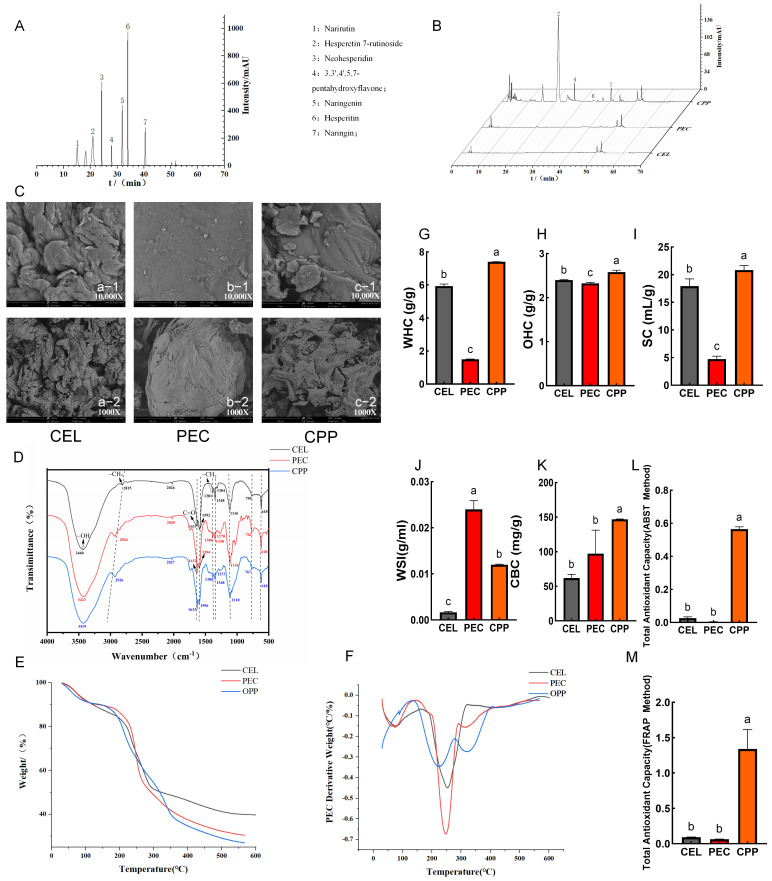
Study on the Composition, Structure, and Functional Properties of CEL, PEC, and CPP. (**A**) Chromatograms of 7 standard flavonoids. (**B**) Chromatograms of flavonoids in CEL, PEC, and CPP (retention time: the time from sample injection to peak retention time). (**C**) SEM analysis of CEL (a), PEC (b), and CPP (c) at magnifications of 10,000× (first row) and 1000× (second row). (**D**) FT-IR analysis of CEL, PEC, and CPP, the same dashed line in Figure indicates identical functional groups in CEL, PEC, and CPP. (**E**) TGA graph. (**F**) DTG graph. (**G**) Water-holding capacity. (**H**) Oil-holding capacity. (**I**) Swelling capacity. (**J**) Water solubility index. (**K**) Cholesterol binding capacity. (**L**) Total antioxidant capacity (ABST method). (**M**) Total antioxidant capacity (FRAP method). Note: Different letters indicate that the difference was significant (*p* < 0.05).

The measured contents of CEL, PEC, lignin, and hemicellulose in CPP (g/100 g dry matter) were 15.62 ± 1.2, 4.27 ± 0.21, 9.43 ± 1.34, and 6.05 ± 0.61, respectively. Furthermore, the SDF-to-IDF ratio in CPP was 15.72 ± 0.95% (Table 1), indicating that CPP is rich in dietary fiber, primarily IDF, consistent with findings by Liu et al. [20] and Llobera et al. [49]. Upon ingestion, these substances may be fermented by gut microbiota in the colon, with the resulting metabolites potentially regulating the composition of intestinal flora [50]. The moisture, protein, fat, ash, total phenolic content, total flavonoid content, and galacturonic acid content of CEL, PEC, and CPP are shown in Table 1. Wen et al. and Qiao et al. have demonstrated that flavonoids alleviated constipation in mice [8,51]. Compared to CEL and PEC, CPP was rich in flavonoids. Figure 1A,B show that five flavonoids were detected in the CPP: narirutin, hesperetin-7-rutinoside, 3,3′,4′,5,7-pentahydroxyflavone, hesperitin, and naringin. Their respective concentrations (mg/g of dry weight) were 29.89 ± 4.75, 155.46 ± 40.19, 3.58 ± 0.80, 8.52 ± 0.89, and 22.89 ± 0.21, respectively.

**Figure 2:**
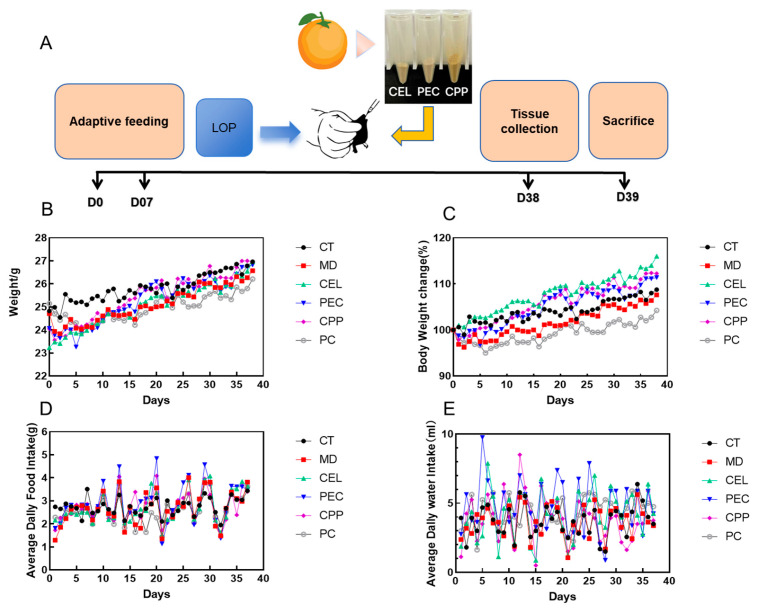
Improvement of physiological conditions in constipated mice by CEL, PEC, and CPP. (**A**) Experimental design. (**B**) Body weight changes. (**C**) Changes in body weight gain rate. (**D**) Changes in food intake. (**E**) Changes in water intake. Note: D0, D07, D38 and D39 represent Day 0, Day 7, Day 38 and Day 39, respectively.

Figure 1C shows the spatial structures of CEL, PEC, and CPP analyzed by SEM at magnifications of 10,000× (first row) and 1000× (second row). At a magnification of 10,000×, CEL Figure 1C(a-1) and CPP Figure 1C(c-1) exhibited curved lamellar structures, whereas PEC particles Figure 1C(b-1) had relatively smooth surfaces with minor protrusions. At a magnification of 1000×, the multilayered, wrinkled, and fluffy states of CEL Figure 1C(a-2) and CPP Figure 1C(c-2) bind readily with water, trapping it within their pores and forming hydrogen bonds or dipole interactions, in contrast to PEC. This conferred excellent water retention and swelling properties, consistent with the conclusions of Zhou et al. and Liu et al. [20,52]. Fourier transform infrared spectroscopy (FTIR) determines the structural and compositional similarity of substances through wavelength, waveform, and peak characteristics (Figure 1D) [53]. The results showed that although CEL, PEC, and CPP exhibited similar absorption peaks for their respective functional groups, the peak shapes differed due to variations in molecular structure and substituents, thereby confirming the structural differences among the three substances. Analysis of weight changes in CEL, PEC, and CPP between 30 and 600 °C revealed greater mass loss in CPP and PEC than in CEL, indicating higher thermal stability in CEL (Figure 1E). All samples exhibited rapid mass loss at 250 °C (Figure 1F). Zeta potential is an indicator of the repulsive and attractive forces between particles and reflects system stability [33]. Compared with PEC and CPP, CEL exhibited the highest absolute zeta potential, indicating greater stability. Particle size analysis revealed that all three materials had submicron-level particle sizes, demonstrating their significant potential as dietary fiber components in fortified foods [54]. PEC had the smallest particle size of the three (Table S3). Zhang et al. suggested that smaller-particle dietary fibers were more effective in alleviating constipation.

The functional properties of dietary fiber are closely related to its physical and chemical characteristics. In the colon, the high solubility of soluble dietary fiber facilitates gel formation that resists dehydration, thereby increasing fecal water content and bulk. Meanwhile, insoluble nutritional fiber stimulates intestinal peristalsis through mechanical action, thereby enhancing fecal bulk and promoting bowel movements [11]. As shown in Figure 1G,I,J, CEL and CPP had a significantly higher WHC and SC than PEC (*p* < 0.05). This may be attributed to their loose, porous structures, which facilitated physical water retention [30]. Higher WHC and SC contribute to increased stool bulk and stimulate intestinal peristalsis [55]. Compared to CEL and CPP, PEC’s high WSI facilitates gel formation, softens stools, and enhances lubrication (*p* < 0.05). Fermentation-derived short-chain fatty acids (SCFAs) lower intestinal pH, promoting probiotic growth and indirectly improving intestinal motility [56]. The strong OHC and CBC of dietary fiber have been reported to reduce intestinal cholesterol levels [57], indicating that CEL, PEC, and CPP may help reduce obesity and constipation, offering health benefits (Figure 1H,K). Citrus flavonoids exhibit strong antioxidant properties [58]. Due to its high flavonoid content, CPP demonstrated excellent antioxidant capacity in vitro (*p* < 0.05) (Figure 1L,M). In summary, CEL’s exceptional WHC and SC may help stimulate intestinal peristalsis. PEC’s good WSI and smaller particle size may facilitate intestinal lubrication and stool softening, while also promoting microbial metabolism to produce relevant metabolites, thereby alleviating constipation. CPP possesses a complex composition. Beyond the WHC and SC conferred by its high insoluble dietary fiber fraction, its flavonoids may also exert laxative effects. Therefore, animal studies are required to validate these hypotheses and determine which component is most effective in relieving constipation.

### 3.2. Improvement of Physiological Status in Constipated Mice by CEL, PEC, and CPP

During the experiment, no abnormal deaths occurred among the six groups of mice. To investigate the efficacy of CEL, PEC, and CPP in alleviating constipation, the therapeutic effects of these substances were evaluated in a LOP-induced constipation model in mice. The experimental design is shown in Figure 2A. Compared with the CT group, body weight and growth rate in the MD group decreased by approximately 2.61% and 1.23%, respectively (see Figure 2B,C), consistent with the findings of Wang et al. [5]. CEL, PEC, and CPP all alleviated weight loss in constipated mice. Body weight and the rate of change in body weight in the positive drug PC group were lower than those in all other groups, possibly due to gastrointestinal irritation caused by the positive drug in this experiment [59]. As shown in Figure 2D,E, food intake and water consumption trends were similar across the groups and exhibited cyclical patterns, consistent with the findings of Gao et al. [60].

### 3.3. Effects of CEL, PEC, and CPP on Defecation Ability, Intestinal Structure, and Barrier Function in Mice

**Figure 3:**
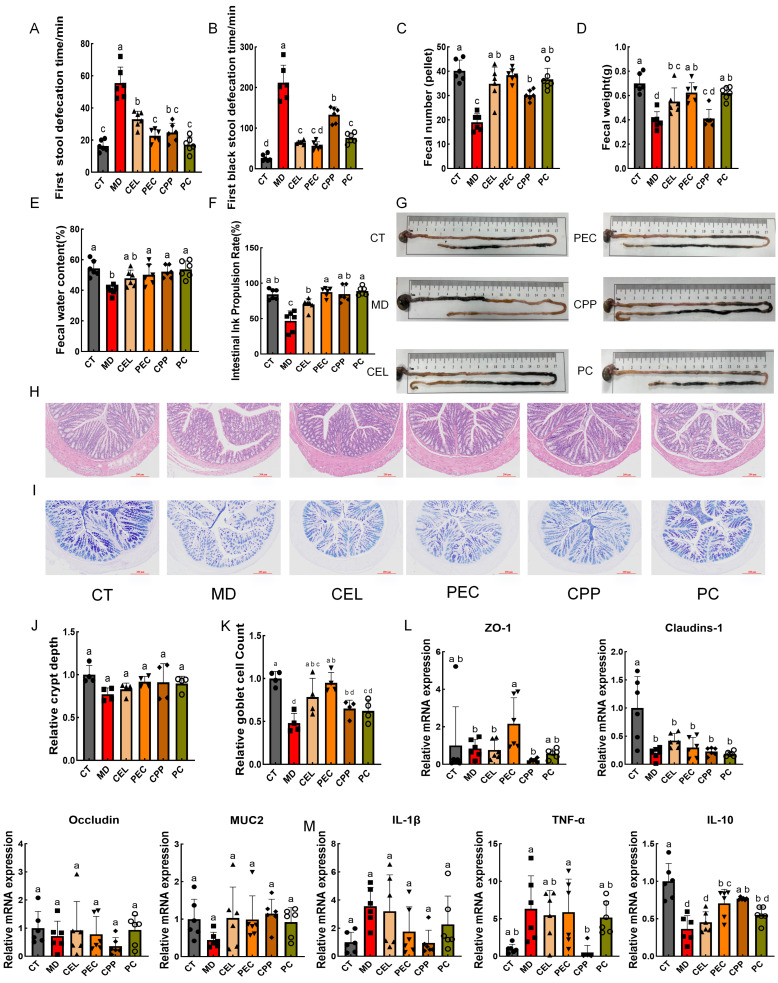
Effects of CEL, PEC, and CPP on Defecation Ability, Intestinal Structure, and Barrier Function in Mice. (**A**) First stool defecation time. (**B**) First black stool defecation time. (**C**) Fecal number. (**D**) Fecal weight. (**E**) Fecal water content. (**F**) Intestinal ink propulsion rate. (**G**) Representative intestinal ink propulsion distance diagram. (**H**) Hematoxylin and eosin (H&E) staining. (**I**), Alcian blue-pink acid (AB-PAS) staining. (**J**) Relative crypt depth. (**K**) Relative goblet cell count. (**L**) Tight junction factor and *MUC-2* mRNA expression levels. (**M**) Inflammatory factor mRNA expression levels. Note: Different letters indicate that the difference was significant (*p* < 0.05). Each scatter point in the bar chart represents a sample, with different groups indicated by different shapes.

As a receptor agonist, LOP primarily induces constipation by inhibiting intestinal peristalsis and water reabsorption. Common symptoms of constipation include prolonged defecation time, incomplete evacuation, and dry, hard stools [61]. Therefore, time to first defecation, fecal volume, weight, and moisture content are the most direct indicators for evaluating constipation models. As shown in Figure 3A–E, compared with the CT group, the MD group was found to increase the time to first defecation and the time to first black stool in mice, while decreasing the total fecal count, fecal weight, and fecal moisture content within six hours (*p* < 0.05). After treatment with PEC, CEL, and CPP, constipation symptoms can be alleviated to some extent. Compared with the CEL group, the PEC group had an approximately 13.38% shorter time to first black stool (*p* < 0.05). Compared with the CPP group, the PEC group reduced the time to first black stool and increased the number and weight of stools within six hours (*p* < 0.05). Additionally, the CEL group had a significantly shorter time to first black stool than the CPP group (*p* < 0.05). Gastrointestinal transit capacity primarily reflects intestinal motility. Adequate dietary fiber supplementation helps soften stools and increase fecal bulk, while also reducing the accumulation of harmful intestinal substances by promoting defecation [62]. As shown in Figure 3F,G, the ink propulsion distance in the MD group decreased significantly (*p* < 0.05). Following interventions with CEL, PEC, and CPP, the intestinal ink propulsion rate increased by approximately 49.48%, 86.48%, and 80.80%, respectively. Compared with the CEL group, the PEC group increased intestinal propulsion rate by approximately 24.76% (*p* < 0.05). Overall, PEC may be superior to CEL and CPP at improving defecation indices and gastrointestinal transit capacity in mice. In summary, CEL’s effect on promoting bowel movements through physical stimulation is weaker than that of PEC. This may be because PEC can be utilized more efficiently by gut microbiota, producing more metabolites that enhance intestinal function. Furthermore, CPP’s laxative effect is more comparable to cellulose. This is because, although it contains both flavonoids and dietary fiber, CPP’s functional ingredients may be diluted at equivalent doses. Its efficacy likely stems primarily from its insoluble dietary fiber fraction. Thus, the effectiveness of CEL, PEC, and CPP in relieving constipation may be closely related to their composition, structure, and functional characteristics. Furthermore, we need to investigate the specific mechanisms underlying their constipation-relieving effects.

Numerous studies indicate that constipation disrupts intestinal structure and barrier function. Intestinal damage impairs peristalsis and fecal transit [40] and also causes crypt injury, intestinal wall thinning, and infiltration of inflammatory factors [63]. Observation of haematoxylin and eosin (H&E)-stained colon sections from mice (Figure 3H,J) revealed that in the CT group, the colonic structure of mice remained intact with well-separated epithelial crypts. In contrast, crypt depth in the MD group decreased by approximately 22.86%. Treatment with CEL, PEC, CPP, and positive drugs demonstrated a certain degree of improvement, with PEC increasing crypt depth by about 19.00%. Colonic goblet cell atrophy may also contribute to constipation to some extent [64]. As shown in Figure 3I,K, the model group exhibited a significant decrease in colonic goblet cell density (*p* < 0.05). In contrast, CEL and PEC partially reversed this effect, with PEC demonstrating particularly pronounced efficacy (*p* < 0.05). Furthermore, tight junction (TJ) factors and mucins, which are located between intestinal epithelial cells, are critical components of the intestinal mechanical barrier. The above histological findings suggest that CEL, PEC, and CPP may alleviate constipation by repairing colonic tissue. Subsequent detection of mRNA expression levels for relevant colonic TJ proteins and mucin *MUC-2* (Figure 3L) revealed that, compared with the MD group, CEL, PEC, and CPP exhibited upregulation of ileal TJ protein (*Claudins*, *Occludin*) and mucin *MUC-2* mRNA expression. Disruption of intestinal barrier function triggers dysbiosis and metabolic imbalance, thereby promoting inflammation and intestinal diseases [5]. Inflammatory cytokines include both pro-inflammatory factors, such as *IL-1β* and *TNF-α*, and anti-inflammatory factors, such as *IL-10*. *IL-1β* recruits neutrophils and promotes the release of inflammatory mediators, whereas *TNF-α* is considered to have tumor necrosis activity [65]. *IL-10* effectively mitigates the effects of inflammation and maintains gut microbiota homeostasis [66]. As shown in Figure 3M, in the MD group, *IL-1β* and *TNF-α* mRNA expression increased, whereas *IL-10* mRNA expression decreased significantly (*p* < 0.05). Meanwhile, CEL, PEC, and CPP demonstrated a specific alleviating effect. Consistent with our results, Zhang et al. and Mo et al. demonstrated that dietary chitin oligosaccharides and burdock root fiber effectively reduced pro-inflammatory factors (*TNF-α* and *IL-1β*) [6,67]. Furthermore, both the PEC and CPP groups exhibited significantly higher *IL-10* mRNA expression than the CEL group (*p* < 0.05). This demonstrated that, as an SDF, PEC may enhance intestinal anti-inflammatory levels more effectively than CEL, which functions as an IDF. This may be due to specific metabolites produced during the thorough fermentation and degradation of PEC in the gut. Furthermore, the anti-inflammatory effects of CPP may stem from the beneficial action of its flavonoid components. In summary, CEL, PEC, and CPP can, to some extent, protect intestinal tissue integrity and mitigate the damaging effects of constipation on the intestinal barrier.

### 3.4. Effects of CEL, PEC, and CPP on Aquaporin Expression and Motility in Mouse Intestines

**Figure 4:**
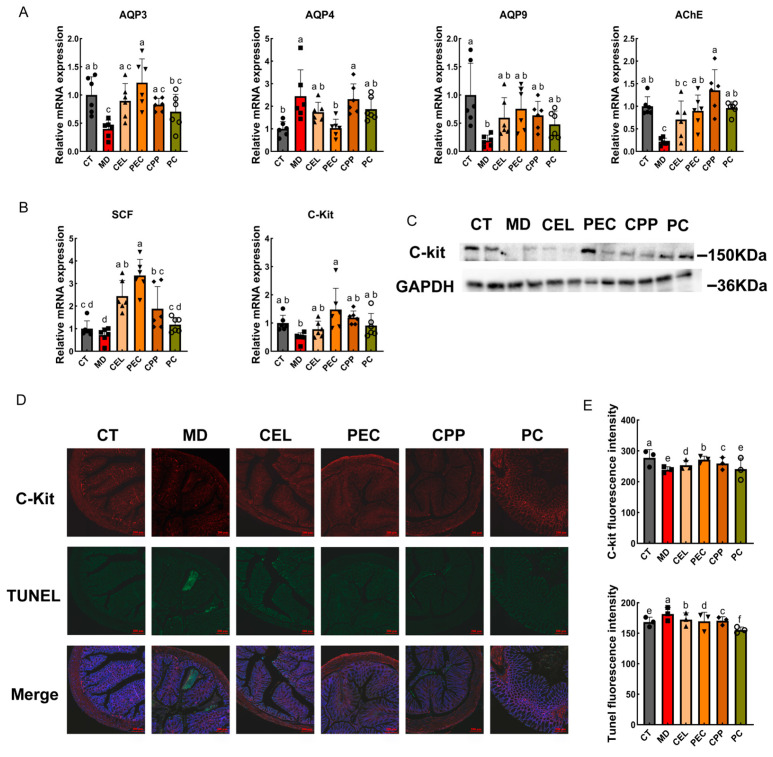
Effects of CEL, PEC, and CPP on aquaporin expression and motility in mice. (**A**) mRNA expression levels of aquaporins and *AChE*. (**B**) mRNA expression levels of *SCF* and *C-kit*. (**C**) Protein expression of C-kit. (**D**) Apoptosis in colonic interstitial cells (ICCs): C-kit-positive ICCs stained red; TUNEL-positive ICCs stained green. (**E**) Fluorescence intensity of C-kit and TUNEL. Note: Different letters indicate that the difference was significant (*p* < 0.05). Each scatter point in the bar chart represents a sample, with different groups indicated by different shapes.

Another cause of constipation is abnormal aquaporin (AQP) expression and impaired ICC apoptosis. Research confirms that numerous AQPs are present in the intestinal mucosa. Within the intestine, AQPs play a crucial role in transmembrane water transport and reabsorption. Abnormal AQP expression can disrupt intestinal water balance and slow down motility. Zhang et al. discovered that AQP3 was essential for regulating fecal water content and that its high expression alleviates constipation symptoms [68]. AQP3 helps regulate the dynamic balance of water across cell membranes, promoting water secretion into the intestinal lumen, thereby softening stool and relieving constipation [69]. AQP9 participates in the synthesis and secretion of mucus, which protects the intestinal surface and facilitates the smooth passage of intestinal contents [70]. Lin et al. also observed that constipation was associated with AQP9 downregulation [71]. Furthermore, the overexpression of AQP4 also caused constipation [72]. For example, Wang et al. found that knocking down the AQP4 gene reduced water absorption in the colon and significantly increased fecal water content, thereby alleviating constipation [8]. Acetylcholinesterase (AChE) promotes intestinal motility by controlling the synthesis and utilization of Acetylcholine (ACh) [6]. Our study (Figure 4A) revealed reduced mRNA expression of *AQP3*, *AQP9*, and *AChE* in the intestines of constipated mice, alongside increased *AQP4* mRNA expression (*p* < 0.05). This finding is consistent with the results reported by Wang et al. and Mo et al. [5,6]. Regarding the aforementioned results, CEL, PEC, and CPP exhibited specific alleviating effects. Among these, PEC significantly increased the expression of *AQP3*, *AQP9*, and *AChE* (*p* < 0.05), whereas it reduced *AQP4* expression (*p* < 0.05). In the gastrointestinal tract, ICCs act as pacemaker cells and are involved in neuromuscular transmission [73]. Reduced or absent ICCs have been observed in the colons of patients with constipation. C-kit, a marker specific to ICCs, binds to stem cell factor (SCF) to help regulate intestinal motility and inflammation, and is closely associated with mesenchymal cell apoptosis [6]. For this experiment, we used C-kit immunolabelling of colon sections and TUNEL staining to detect apoptotic ICCs. The results shown in Figure 4B–E indicated that, compared with the CT group, *SCF* and *C-Kit* mRNA expression levels in constipated mice decreased by approximately 28.28% and 52.2%, respectively. C-Kit protein expression decreased, and red fluorescence (C-Kit) expression in colon tissue diminished (*p* < 0.05). Concurrently, green fluorescence representing ICC apoptosis increased (*p* < 0.05). These results suggested that reduced *SCF* and *C-Kit* expression may enhance ICC apoptosis, thereby exacerbating the occurrence of constipation. Conversely, CEL, PEC, and CPP effectively reversed this phenomenon (*p* < 0.05). Compared with the CEL and CPP groups, the PEC group exhibited significantly higher red fluorescence and lower green fluorescence (*p* < 0.05). These findings confirm that these substances may mediate ICC apoptosis via the SCF/C-Kit pathway. PEC showed more substantial effects compared to CEL and CPP.

### 3.5. Effects of CEL, PEC, and CPP on Intestinal Oxidative Stress and Apoptosis in Mice

**Figure 5:**
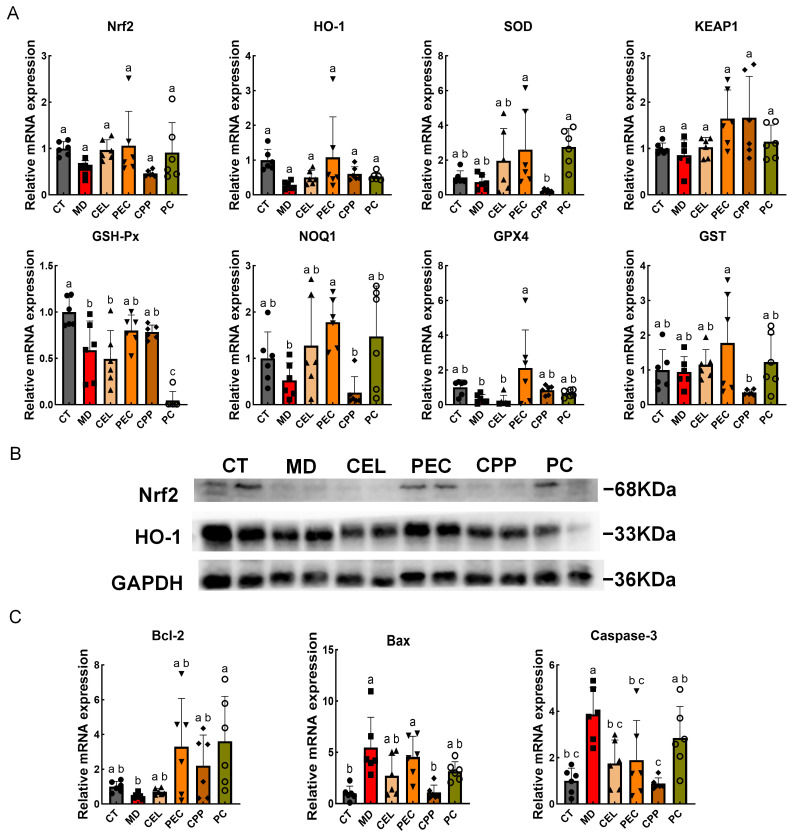
Effects of CEL, PEC, and CPP on oxidative stress and apoptosis in mouse intestines. (**A**) mRNA expression levels of the Nrf2-HO-1 pathway in the colon. (**B**) Protein expression of the Nrf2-HO-1 pathway in the colon. (**C**) mRNA expression levels of apoptosis-related factors. Note: Different letters indicate that the difference was significant (*p* < 0.05). Each scatter point in the bar chart represents a sample, with different groups indicated by different shapes.

Oxidative stress plays a pivotal role in the development of constipation by disrupting intestinal homeostasis and inducing apoptosis in mesenchymal cells. Studies indicate that elevated oxidative stress in patients with constipation and in animal models is closely associated with increased ICC apoptosis. Therefore, alleviating oxidative stress and the resulting apoptosis represents an effective strategy for relieving constipation [2,74]. Yao et al. demonstrated that the Nrf2/HO-1 pathway protects ICCs from oxidative stress damage, thereby alleviating intestinal motility disorders [48]. Under oxidative stress conditions, studies indicated that the transcription factor Nrf2 modulated the expression of stress-related proteins and enzymes involved in metabolism and detoxification. This induced the production of various cytoplasmic antioxidant factors, including HO-1, SOD, and GSH-Px. We therefore examined the gene expression of the Nrf2-HO-1 pathway and its associated antioxidant factors (Figure 5A). Research findings indicate that constipation is associated with decreased mRNA expression of antioxidant factors *Nrf2*, *HO-1*, *SOD*, *KEAP1*, *GSH-Px*, *NQO1*, and *GPX4* in the colon. However, CEL, PEC, and CPP partially mitigated this trend. Compared with the model group, PEC significantly increased mRNA expression levels of *NQO1* and *GPX4* (*p* < 0.05). Furthermore, Figure 5B showed that PEC increased Nrf2 protein expression in colon tissue of constipated mice and simultaneously activated its target gene, HO-1, thereby exerting antioxidant effects.

Li et al. found that intestinal oxidative stress invariably occurs alongside apoptosis [75]. Apoptosis is closely linked to an imbalance in Bcl-2 and Bax expression. Bcl-2 has anti-apoptotic properties, whereas Bax is a well-known pro-apoptotic factor. Disruption of the Bcl-2/Bax balance induces apoptosis [76]. We therefore measured the mRNA expression of the anti-apoptotic factor *Bcl-2*, as well as the pro-apoptotic factors *Bax* and *Caspase-3*, in mouse intestines (Figure 5C). As expected, constipation caused an imbalance in *Bcl-2*/*Bax* expression in mice, accompanied by increased expression of the pro-apoptotic factor *caspase-3* (*p* < 0.05). Compared with the MD group, the CEL, PEC, and CPP groups all effectively reversed *caspase-3* mRNA expression (*p* < 0.05). Additionally, PEC significantly increased *Bcl-2* mRNA expression (*p* < 0.05). Therefore, our findings suggest that PEC may alleviate constipation by modulating apoptosis through the SCF/C-Kit and Nrf2/HO-1 pathways.

### 3.6. Effects of CEL, PEC, and CPP on Gut Microbiota Composition in Mice

**Figure 6:**
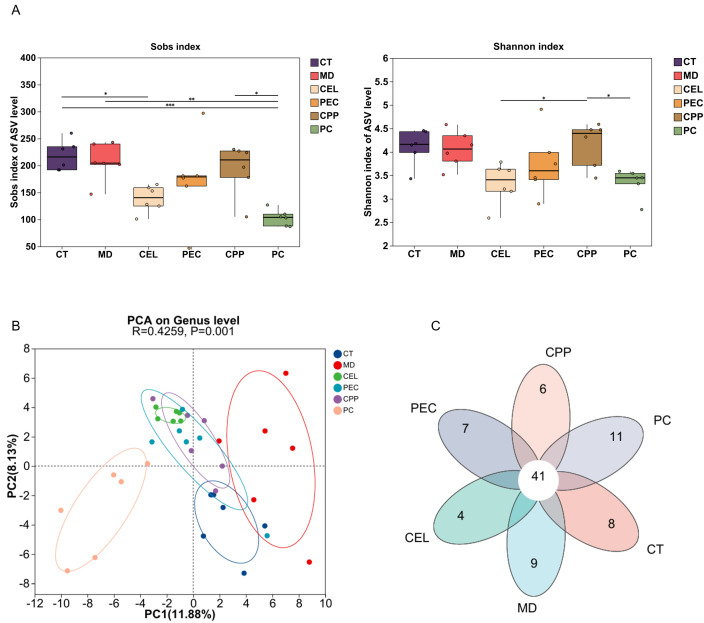

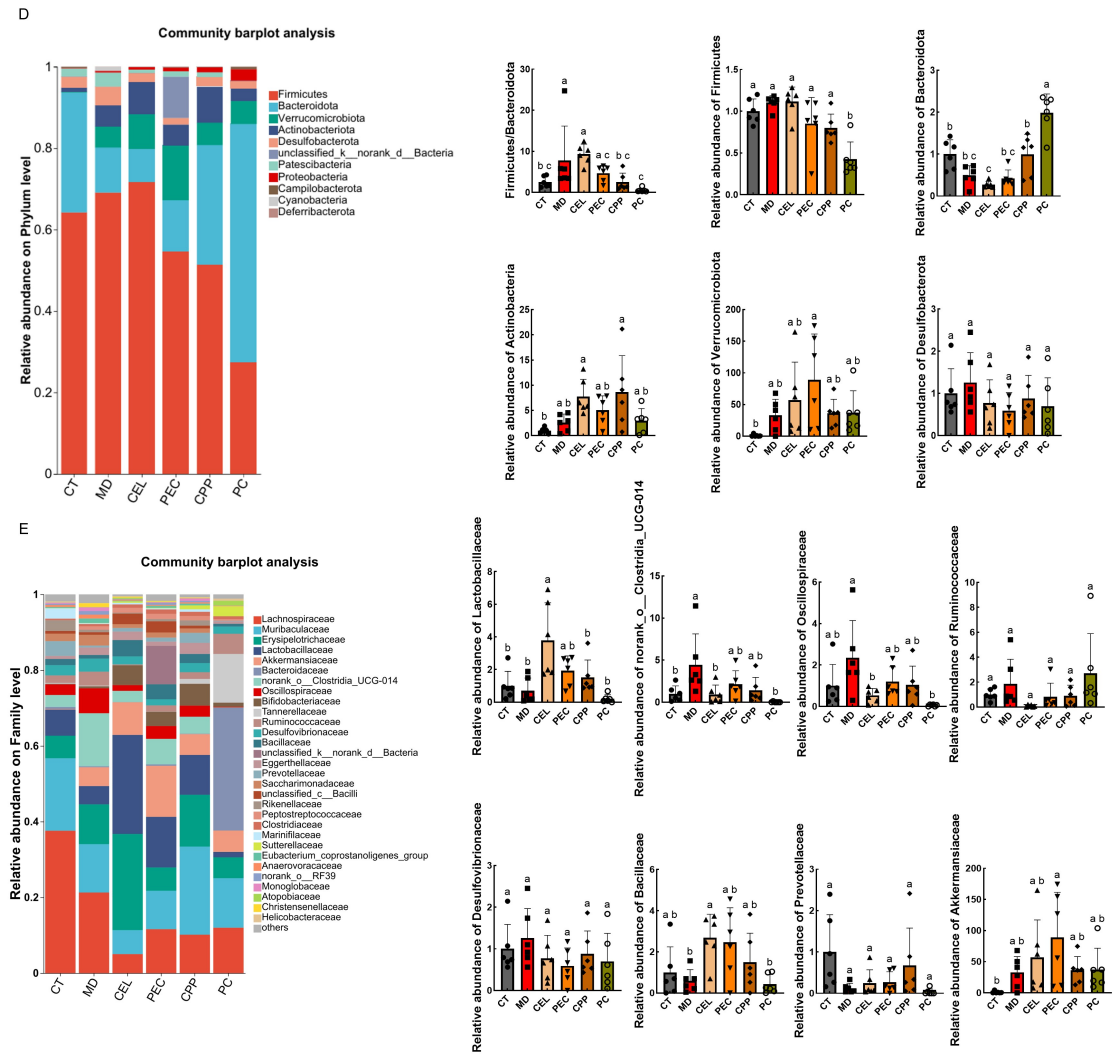

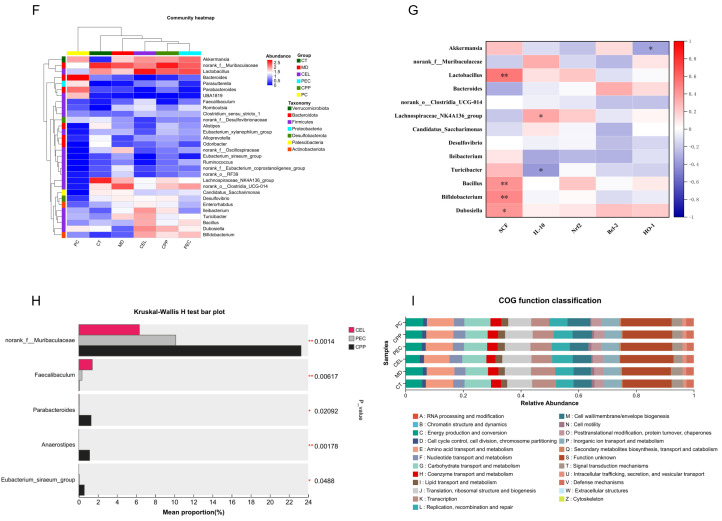
Effects of CEL, PEC, and CPP on gut microbiota diversity and differential analysis in constipated mice. (**A**) α-diversity analysis. (**B**) PCA. (**C**) Venn diagram. (**D**) Gut microbiota analysis at the phylum level. (**E**) Gut microbiota analysis at the family level. (**F**) Heatmap of the top 30 genus abundances. (**G**) Correlation analysis. (**H**) Multi-group comparison analysis. (**I**) COG Function Prediction. Note: When *p* < 0.05 between groups, the results were considered statistically significant. * *p* < 0.05; ** *p* < 0.01; *** *p* < 0.001; ns, not significant. Different letters indicate significant differences (*p* < 0.05). Each scatter point in the bar chart represents a sample, with different groups indicated by different shapes.

Disruption of the gut microbiota balance is closely associated with the onset of constipation [77]. This study used 16S rRNA gene sequencing analysis to investigate the potential impact of CEL, PEC, and CPP on the gut microbiota of constipated mice. Figure 6A shows that, compared with the CT group, the richness and diversity of gut microbes in constipated mice did not change significantly (*p* > 0.05). Furthermore, neither PEC nor CEL affected the Sobs and Shannon indices of gut microbial ASV in these mice. This is consistent with the findings of Yang et al. [11]. PCA and Venn diagram results (Figure 6B,C) revealed significant differences in the gut microbiota of constipated mice compared to normal mice (R = 0.4259, *p* = 0.001). However, the gut microbiomes of mice that consumed CEL, PEC, or CPP were more similar to those of control mice. These results suggest that alterations in the gut microbiota may mediate the laxative effects of these three substances on constipated mice.

Figure 6D shows the phylum level. The five most abundant phyla are *Firmicutes*, *Bacteroidetes*, *Verrucomicrobia*, *Actinobacteria,* and *Desulfobacterota*. The *Firmicutes* and *Bacteroidetes* phyla exhibited the highest relative abundances, collectively accounting for over 80% of the total relative abundance. Elevated proportions of *Firmicutes* and *Bacteroidetes* are closely associated with constipation and obesity [2]. Compared with the CT group, the MD group showed a higher F/B ratio (*p* < 0.05). In contrast, the PEC and CPP groups reduced Firmicutes abundance by approximately 22.91% and 27.53%, respectively, thereby mitigating the increase in F/B. The phylum *Verrucomicrobia* primarily comprises the beneficial bacterium *Akkermansia*. Numerous experiments have shown that alleviating intestinal inflammation is closely associated with increased abundance of *Verrucomicrobia* [78,79]. *Desulfobacterota* is not a direct pathogen, but its increased abundance may contribute to the development of colitis and irritable bowel syndrome (IBS) [80]. Additionally, its abundance is higher in individuals with constipation than in healthy individuals [81]. The results indicate that, compared with the MD group, the CEL, PEC, and CPP groups showed a tendency toward increased relative abundance of *Verrucomicrobia* and reduced relative abundance of *Desulfobacterota*. Among these, the PEC group exhibited the most pronounced effect, increasing *Verrucomicrobia* content by approximately 170% and decreasing *Desulfobacterota* content by approximately 53.04%.

At the family level (Figure 6E), although no significant differences were observed compared with the CT group, MD group mice tended to have decreased abundance of *Lactobacillaceae*, *Prevotellaceae*, and *Bacillaceae*. In contrast, *Oscillospiraceae*, *Ruminococcaceae*, and *Desulfovibrionaceae* showed a tendency toward increased abundance. Additionally, the abundance of *norank_o_clostridia_ucg-014* significantly increased in model group mice (*p* < 0.05). Increased abundance of *Lactobacillaceae* has been shown to alleviate constipation [46]. *Prevotellaceae* can digest complex carbohydrates and produce SCFAs; their abundance is inversely correlated with abdominal pain. Deng et al. found that *Desulfovibrionaceae* and *Oscillospiraceae* were positively correlated with serum IL-1β and TNF-α levels, and negatively correlated with fecal water content and SCFA levels [62]. *Akkermansiaceae* facilitate reduced inflammatory cell infiltration and increased thickness of the intestinal epithelium and mucus layer, thereby maintaining intestinal barrier function and integrity [63]. Compared with the MD group, the CEL, PEC, and CPP groups increased the abundance of *Akkermansiaceae* by approximately 73.33%, 171.19%, and 10.77%, respectively, with PEC showing the most tremendous increase.

Heatmap analysis (Figure 6F) of the dominant genera (top 30) revealed that, compared with the CT group, the relative abundances of *Muribaculaceae*, *Lactobacillus*, and *Dubosiella* decreased by approximately 8.59%, 15.02%, and 59.02%, respectively, in the MD group. Meanwhile, the relative abundances of *Desulfovibrio* and *Candidatus_Saccharimonas* increased by approximately 13.44% and 2.18%, respectively. CEL, PEC, and CPP interventions can restore the relative abundance of *Lactobacillus* and *Dubosiella*. Liu et al. demonstrated that *Dubosiella* is particularly effective in reducing oxidative stress, improving vascular endothelial function, and redistributing the gut microbiota [82]. Butorac et al. found that *Candidatus Saccharimonas* is associated with the development of inflammatory bowel disease, and that increased abundance of this species increases the risk of intestinal inflammation [83]. Compared with the MD group, the abundance of *Akkermansia* increased by approximately 34%, whereas the abundance of *Desulfovibrio* decreased by approximately 26% in the PEC group. Compared with the CEL and CPP groups, *Akkermansia* abundance increased by approximately 11.09% and 13.03%, respectively, whereas *Desulfovibrio* abundance decreased by approximately 23.99% and 26.27%, respectively. To further investigate the association between the gut microbiota and constipation, we performed Spearman’s rank correlation analysis between the gut microbiota and constipation-related indicators (Figure 6G). As shown in the figure, *Lactobacillus*, *Bacillus*, and *Bifidobacterium* showed significant positive correlations with *SCF* expression (*p* < 0.01), whereas *Dubosiella* also showed a positive correlation (*p* < 0.05). These findings suggest that these microorganisms may help alleviate constipation symptoms.

Analysis of the differential microbiota among the CEL, PEC, and CPP groups revealed a significant difference in *Muribaculaceae* abundance (Figure 6H, *p* = 0.0014). Cao et al. previously demonstrated that *Muribaculaceae* were crucial for the degradation of complex carbohydrates and the production of short-chain fatty acids [2]. The physiological mechanisms governing the gut microbiota were predicted using PICRUSt2, with relevant metabolic pathway information obtained from the KEGG database. COG function prediction results indicate impaired carbohydrate transport and metabolic function in the MD group, which were mitigated by CEL and PEC interventions (Figure 6I). In summary, the intake of CEL, PEC, and CPP can modulate the gut microbiota to a certain extent. Comparisons of key microbial communities at the phylum, family, and genus levels revealed that PEC better regulates the balance between beneficial and harmful gut microbes than CEL and CPP. Furthermore, differences in the rates at which gut microbes degrade and utilize CEL, PEC, and CPP likely lead to distinct metabolic byproducts, thereby explaining the variations in constipation-relieving effects.

### 3.7. Effects of CEL, PEC, and CPP on Mouse Metabolites

**Figure 7:**
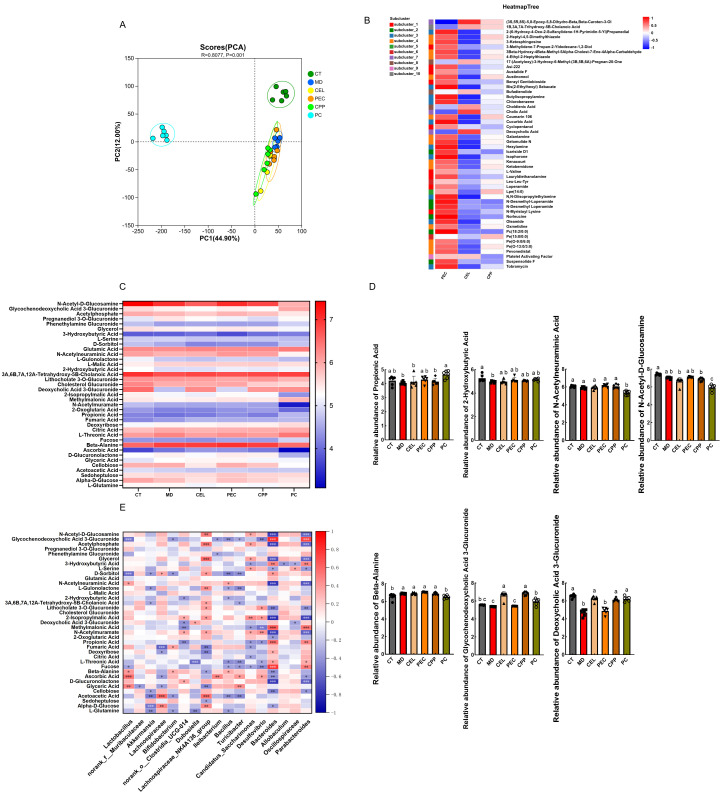
Metabolomic analysis of the mouse gut. (**A**) PCA. (**B**) Heatmap. (**C**) Differences in Carbohydrate Metabolites. (**D**) Comparison of key carbohydrate metabolites. (**E**) Correlation analysis between carbohydrate metabolites and gut microbiota. Note: When *p* < 0.05 between groups, the results were considered statistically significant. * *p* < 0.05; ** *p* < 0.01; *** *p* < 0.001; ns, not significant. Different letters indicate significant differences (*p* < 0.05). Each scatter point in the bar chart represents a sample, with different groups indicated by different shapes.

PCA revealed significant differences in metabolite composition between groups (Figure 7A, R = 0.8077, *p* = 0.001). Under the conditions of the OPLS-DA model (VIP ≥ 1, Fold Change = 1, *p*-value < 0.05), differential metabolites were identified between each pair of groups across the six samples. Volcano plot results (Figure S2) revealed that, compared with the MD group, the CT group exhibited 517 differentially expressed metabolites. In contrast, the CEL group showed 508, the PEC group 260, the CPP group 282, and the PC group 629. Differential Metabolite Statistics Chart revealed that, compared to the CT group, the MD group exhibited elevated levels of opioid substances (morphine, oxymorphone) and the loperamide metabolite N-Desmethyl loperamide (*p* < 0.05). It has been demonstrated that opioids act on the central nervous system by slowing intestinal motility and prolonging the retention time of food residues in the intestinal lumen. This increases water absorption and simultaneously interferes with the defecation reflex, thereby weakening the urge to defecate [11]. Compared with the MD group, the CEL group reduced the level of the opioid metabolite (R)-apocodeine (*p* < 0.05), which similarly weakens intestinal motility. In contrast, the PEC group increased the abundance of the metabolites Gomisin D and Rosamicin (*p* < 0.05). Gomisin D scavenges ABTS (+) radicals, thereby reducing oxidative stress [84] and partially explaining PEC’s favorable performance in antioxidant stress pathways. Rosamicin exhibits potent anti-inflammatory and antibacterial activity [85]. Compared with the MD group, the CPP group significantly increased the levels of flavonoids, such as Hesperidin and Naringin, in intestinal metabolites (*p* < 0.05). Citrus flavonoids have been shown to alleviate constipation [8,51]. To further investigate the effects of CEL, PEC, and CPP metabolites on constipation relief, a heatmap was generated using the top 50 differential metabolites in abundance for each group. The clustering heat map depicts samples in columns and differential metabolites in rows, with color intensity indicating expression levels. As shown in Figure 7B, the results indicated that the PEC group exhibited higher metabolite diversity and greater abundance, suggesting that PEC may be effectively metabolized in the mouse intestine. Compared with the PEC group, the relative abundance of most metabolites decreased in the CEL group, further indicating that CEL, as an insoluble dietary fiber, is difficult for the gut microbiota to metabolize and utilize. The relative abundance changes in metabolites in the CPP group fell between those of the two groups, possibly due to its simultaneous presence of both CEL and PEC components. Specifically, compared with the CEL and CPP groups, PEC increased the abundance of Tobramycin and Oleamide. Tobramycin has been reported to alleviate intestinal inflammation [86], while Oleamide exhibits potent anti-inflammatory and antioxidant activity [87]. In contrast, the CEL group showed increased abundance of deoxycholic acid and cholic acid compared to the PEC and CPP groups. However, existing literature indicates that excessively high concentrations may increase cytotoxicity and inflammatory responses [88]. Although this experiment did not directly measure related toxicity, alterations in relevant metabolites reveal such potential risks. In summary, the constipation-relieving effects of CEL, PEC, and CPP may be mediated by their metabolic by-products. Differences in these byproducts account for variations in constipation relief. Compared to the CEL and CPP groups, the PEC group may be more readily utilized by the gut microbiota due to its smaller particle size and superior solubility. This increases the diversity and abundance of metabolic by-products, as well as the levels of beneficial by-products, further demonstrating that PEC is more effective than CEL and CPP in relieving constipation.

Previous sections have discussed differences in carbohydrate metabolism among members of the gut microbiota. Therefore, relevant carbohydrate metabolites were screened based on KEGG functional pathways. A clustering heatmap was generated, with columns representing samples, rows representing differential metabolites, and color intensity reflecting expression levels (Figure 7C). Relative carbohydrate content is shown in Figure S2. The results revealed that 37 carbohydrate metabolites were identified in all groups, including acetyl phosphate, glycerol, L-serine, and D-sorbitol. Comparedto the control group, the MD group exhibited a reduced overall abundance of carbohydrate metabolites. However, the CEL, PEC, and CPP groups mitigated this reduction, with the PEC group showing the most significant increase in carbohydrate metabolite abundance. These included glycochenodeoxycholic acid 3-glucuronide, acetylphosphate, N-acetyl-d-glucosamine, 2-isopropylmalic acid, N-acetylneuraminic acid, Methylmalonic acid, 2-hydroxybutyric acid, Propionic acid, Cholesterol Glucuronide, Phenethylamine Glucuronide, and L-gulonolactone. This indicates that they may be broken down and utilized more efficiently by gut microbiota or host metabolic enzyme systems. Furthermore, compared to the CEL and CPP groups (Figure 7D), the PEC group increased the levels of short-chain fatty acids (2-hydroxybutyric acid and Propionic acid), N-acetyl-d-glucosamine, N-acetylneuraminic acid, and β-Alanine (5.10 ± 0.21, 4.29 ± 0.25, 7.07 ± 0.07, 6.14 ± 0.15, 7.02 ± 0.05). SCFAs serve as a link between the microbiota, redox signaling, and host metabolism. Our research indicates that PEC may regulate Nrf2 by increasing SCFA levels, thereby maintaining intestinal redox balance [89]. N-acetyl-d-glucosamine contributes to enhanced intestinal barrier function. As a precursor to carnosine, β-Alanine modulates intestinal neuronal excitability and promotes peristalsis. Research demonstrates that β-alanine and carnosine enhance antioxidant capacity and ameliorate metabolic dysregulation in diabetes and related disorders [90]. PEC can also moderately increase bile acid levels, specifically glycochenodeoxycholic acid 3-glucuronide and deoxycholic acid 3-glucuronide. Yin et al. observed lower bile acid levels in the feces of constipated patients and concluded that appropriate bile acid supplementation is a viable therapeutic strategy for treating constipation [91]. These findings further explain why PEC is significantly more effective than CEL and CPP in relieving constipation.

To gain deeper insights into the mechanisms by which CEL, PEC, and CPP alleviate constipation, Spearman’s rank correlation analysis was conducted between the gut microbiota and carbohydrate-related metabolites. The results are shown in Figure 7E. At the genus level, *Lactobacillus* showed a positive correlation with N-acetylneuraminic acid (*p* < 0.05), *Dubosiella* showed a positive correlation with Deoxycholic acid 3-glucuronide (*p* < 0.05), and *Candidatus_Saccharimonas* showed a negative correlation with Propionic acid (*p* < 0.05). The results suggested a possible correlation between the abundance of microorganisms regulated by CEL, PEC, and CPP (such as *Lactobacillus*, *Dubosiella*, and *Candidatus Saccharimonas*) and the levels of Deoxycholic Acid 3-Glucuronide and Propionic acid. Additionally, *Desulfovibrio* showed an inverse relationship with Propionic acid (*p* < 0.05) and glycochenodeoxycholic acid 3-glucuronide (*p* < 0.01). This suggested that PEC may influence the levels of Propionic acid and glycochenodeoxycholic acid 3-glucuronide by regulating *Desulfovibrio* levels. The results indicate that changes in gut microbiota are closely associated with the abundance of metabolic products. The effects of CEL, PEC, and CPP on gut microbial abundance may similarly influence the abundance of metabolic products. Compared with CEL and CPP, PEC effectively modulates the abundance of relevant microbial populations while increasing both the diversity and the content of beneficial metabolites. Thereby delivering the optimal constipation-relieving effect.

The composition, structure, and functional characteristics of CEL, PEC, and CPP significantly influence their efficacy in alleviating constipation. Examining differences in their physicochemical properties is crucial for understanding their mechanisms of action and therapeutic effects. The results obtained from this mouse model of LOP-induced constipation cannot be directly extrapolated to humans. Additionally, some analyses relied primarily on mRNA expression levels and correlation data, with no further validation at the protein level. Furthermore, as a preliminary screening of functional substances, this study employed equal doses of CEL, PEC, and CPP to ensure comparability. This approach may have obscured the assessment of synergistic effects among CPP’s diverse components and lacked comparative analysis of the actions of relevant functional constituents. Therefore, to provide reliable translational medical evidence for developing functional dietary fiber products, future research urgently requires randomized controlled clinical trials in humans to validate their efficacy and safety in relieving constipation. Concurrently, dose optimization studies should be conducted to explore potential mechanisms underlying the multi-component interactions within CPP.

## 4. Conclusions

This study systematically characterized the composition, structure, and functional properties of CEL, PEC, and CPP and compared their effects on relieving constipation. Results indicate that cellulose exhibits stable properties, with its constipation-relieving effects primarily depending on its physical characteristics. At equivalent doses, CPP contains functional components at relatively low concentrations and is composed mainly of insoluble dietary fiber, resulting in a weaker overall laxative effect. In contrast, PEC, owing to its smaller particle size and superior water solubility, more effectively modulates gut microbiota and elevates beneficial metabolite levels. Additionally, PEC demonstrated superior effects in regulating aquaporin gene expression, activating SCF/C-kit and Nrf2/HO-1 signaling pathways, and reducing ICC apoptosis. These findings suggest that PEC has promising potential for functional food development. However, further investigation is needed to assess its effects on humans, including long-term safety evaluations and determination of appropriate dosages.

## Figures and Tables

**Table 1:** The content and proportion of each component in CEL, PEC, and CPP.

Sample	Moisture (%)	Ash (%)	Crude Protein (%)	Crude Fat (%)	Total Flavonoids (mg/mL)	Total Phenols (mg/g)	Galacturonic Acid Content (%)	SDF/IDF (%)
CEL	11.19 ± 0.22	0.35 ± 0.03	0.28 ± 0.025	0.053 ± 0.015	0.48 ± 0.041	61.86 ± 5.33	/	/
PEC	9.51 ± 0.69	0.45 ± 0026	0.38 ± 0.035	0.27 ± 0.047	0.075 ± 0.041	97.29 ± 34.03	74.43 ± 4.63	/
CPP	11.81 ± 0.71	3.63 ± 0.078	2.43 ± 0.10	1.19 ± 0.053	77.82 ± 0.19	146.84 ± 0.6	3.18 ± 0.062	15.72 ± 0.95

## Data Availability

The original contributions presented in the study are included in the article/Supplementary Materials; further inquiries can be directed to the corresponding authors.

## Supplementary Materials

The following supporting information can be downloaded at https://www.mdpi.com/article/10.3390/foods15020240/s1: Figure S1:  Process Flow Chart for CEL and PEC Preparation; Figure S2: Volcano Plot and Differential Metabolite Statistics Chart; Figure S3: Western Blot Original Image; Table S1: Sample size for each experiment; Table S2: Primer Information; Table S3: zeta potential and particle size of CEL, PEC, and CPP; Table S4: Relative content of carbohydrate metabolites.
