# Supplementary material for: Comparative Study of the Effectiveness of Cellulose, Pectin and Citrus Peel Powder in Alleviating Loperamide-Induced Constipation
Source: Foods. 2026 Jan 9;15(2):240. doi: 10.3390/foods15020240 (PMC12840159; doi:10.3390/foods15020240)
Supplement: Supplementary file 1 [file foods-15-00240-s001.zip › foods-4058443-supplementary.pdf]

Supplement data

Supplement Table S1. Sample size for each experiment

Experimental group	Initial number of animals	Gut Microbiome Analysis	Fecal Metabolites analysis	Histopathological Staining（H&E and AB-PAS）	Immunofluorescence staining
CT	6	6	6	4	3
MD	6	6	6	4	3
CEL	6	6	6	4	3
PEC	6	6	6	4	3
CPP	6	6	6	4	3
PC	6	6	6	4	3
Total	36	36	36	24	18

Supplement Table S2. Primer Information

Gene	forward primer	reverse prime	BP value	Serial Number	Amplification efficiency	R^2 values
β-actin	GGCTGTATTCCCCTCCATCG	CCAGTTGGTAACAATGCCATGT	154	NM_007393.5	96%	0.9967
IL-1β	GGGCCTCAAAGGAAAGAATC	TACCAGTTGGGGAACTCTGC	183	NM_008361.4	95%	0.9966
TNF-α	ACACCGAGATTTCCTTCAAACTG	CCATCTAGGGTTATGATGCTCTTCA	88	NM_024406.4	105%	0.9951
IL-10	GCTCTTACTGACTGGCATGAG	CGCAGCTCTAGGAGCATGTG	105	NM_010548.2	109%	0.9816
ZO-1	ATAGGAGTGCAAGCAGGGAGA	CCATCTCTTGCTGCCAAACTATC	190	NM_001417367.1	103%	0.9961
Occludin	TGAAAGTCCACCTCCTTACAGA	CCGGATAAAAAGAGTACGCTGG	128	NM_001360536.1	94%	0.9951
Claudin-1	GACTGTGGATGTCCTGCGTTTC	CAATTACCATCAAGGCTCGGG	114	NM_016674.4	97%	0.9942
MUC2	ACATGCCTAATGGCTGCTGT	CTGGGCAGAGTACATGGCAA	167	NM_023566.4	99%	0.9950
AQP3	GGGCCTTTGCCAACAATGAG	CGGCTGTGCCTATGAACTGA	126	NM_016689.2	98%	0.991
AQP4	AAGACCTTTGCTCTGGGCATC	GGAATGTCCACACTTAGACACAG	104	NM_001308641.1	103%	0.996
AQP9	AACAGGCTGCCAAGAGAGGT	GGCCATGAGTCCGTCATAGTA	577	NM_001271843.1	101%	0.9943

AChE	GCCGCCTCAGCAGACAC	GAAAAGCTGAGACTGGGCCT	225	NM_001290010.1	106%	0.9931
SCF	TGCGGGAATGTGACTGATAATG	CCGGCGACATAGTTGAGGGTTATC	95	NM_013598.4	103%	0.9957
C-Kit	GATCTGCTCTGCGCTCTGTTGG	AACTCTGATTGTGCTGGATGG	113	NM_021099.3	94%	0.9961
Nrf2	ACTACAGTCCCAGCAGAGTGAT	TCACACACTTTCTGCGTGCT	109	NM_001399226.1	103%	0.9908
HO-1	CAGAGCCGTCTCGAGCATAG	CAAATCCTGGGGCATGCTGT	108	NM_010442.2	106%	0.9961
SOD	GAACAATCTCAACGCCACCG	CTCCAGCAACTCTCCTTTGGG	181	NM_013671.3	96%	0.9992
KEAP1	GGCGAGTAGAGGTAGGGGTC	TAGGGGCCCCGCCAT	133	NM_001110305.1	108%	0.9969
GSH-Px	CTGCTGCACTCTACCAGACC	GGTTCGAGCTGCTGTCCAC	81	NM_008178.3	101%	0.9953
NQO1	AGAGAGTGCTCGTAGCAGGA	CAGGATGCCACTCTGAATCG	179	NM_008706.5	104%	0.9950
GPX4	CGCCAAAGTCCTAGGAAACG	ATGCACACGAAACCCCTGTA	278	NM_001037741.4	99%	0.9967
GST	TATACAGGTCGGCGATGACAG	TCTCAGTCTTCAAATGACCAGGA	222	NM_001347489.2	93%	0.9947
Bcl-2	CCTGTGGATGACTGAGTACCTG	AGCCAGGAGAAATCAAACAGAGG	123	NM_009741.5	101%	0.9960
Bax	GCCTTTTTGCTACAGGGTTTCAT	TATTGCTGTCCAGTTCATCTCCA	151	NM_001411994.1	105%	0.9939
Caspase-3	GAGCTTGGAACGGTACGCTA	GCGAGATGACATTCCAGTGC	224	NM_001284409.1	109%	0.9850

Supplement Table S3. zeta potential and particle size of CEL, PEC, and CPP

**Sample Name**	**Zeta potential (mV)**	**Particle size (μm)**
Cellulose	30.23±0.97	4.48±0.09
Pectin	19.83±0.82	2.83±0.06
Citrus peel powder	26.4±1.52	3.98±0.05

Supplement Table S4. Relative content of carbohydrate metabolites

Metabolite	CT	MD	CEL	PEC	CPP	PC
**N-Acetyl-D-Glucosamine**	7.43±0.10	7.02±0.10	6.63±0.44	7.07±0.07	6.84±0.21	5.93±0.36
**Glycochenodeoxycholic Acid 3-Glucuronide**	5.57±0.03	5.38±0.02	5.23±0.05	5.41±0.02	5.30±0.09	6.00±0.06
**Acetylphosphate**	5.97±0.03	5.86±0.02	5.71±0.03	5.90±0.04	5.79±0.11	5.46±0.02
**Pregnanediol 3-O-Glucuronide**	5.04±0.58	5.48±0.20	5.29±0.48	5.14±0.30	4.94±0.49	5.30±0.19
**Phenethylamine Glucuronide**	4.73±0.17	4.44±0.27	4.50±0.43	4.44±0.22	4.46±0.26	4.86±0.67
**Glycerol**	5.55±0.03	5.42±0.03	5.34±0.03	5.46±0.03	5.40±0.09	5.18±0.02
**3-Hydroxybutyric Acid**	3.87±0.17	3.90±0.13	4.04±0.12	3.74±0.10	4.03±0.10	4.16±0.10
**L-Serine**	4.84±0.13	4.87±0.07	4.78±0.15	4.80±0.11	4.76±0.09	4.69±0.11
**D-Sorbitol**	4.73±0.29	4.54±0.11	4.22±0.24	4.29±0.23	4.30±0.25	4.75±0.15
**Glutamic Acid**	6.25±0.36	6.23±0.14	6.30±0.16	6.23±0.09	6.27±0.04	6.38±0.14
**N-Acetylneuraminic Acid**	6.06±0.11	5.90±0.11	5.93±0.19	6.14±0.15	6.07±0.23	5.36±0.25
**L-Gulonolactone**	5.14±0.30	4.78±0.28	4.72±0.24	4.87±0.25	4.83±0.22	4.67±0.21
**L-Malic Acid**	5.42±0.11	5.63±0.18	5.66±0.17	5.66±0.15	5.70±0.08	5.54±0.21
**2-Hydroxybutyric Acid**	5.26±0.29	4.95±0.09	4.97±0.17	5.10±0.21	5.05±0.05	5.15±0.10
**3A,6B,7A,12A-Tetrahydroxy-5B-Cholanoic Acid**	7.02±0.04	6.76±0.07	6.85±0.13	6.76±0.14	6.82±0.13	6.90±0.15
**Lithocholate 3-O-Glucuronide**	6.17±0.07	6.13±0.10	6.09±0.13	6.00±0.21	6.15±0.16	5.89±0.11
**Cholesterol Glucuronide**	6.28±0.55	5.95±0.20	5.97±0.36	6.02±0.19	5.68±0.20	5.60±0.32
**Deoxycholic Acid 3-Glucuronide**	6.52±0.21	4.71±0.37	6.25±0.29	4.89±0.36	6.14±0.18	6.22±0.28
**2-Isopropylmalic Acid**	5.39±0.21	5.18±0.08	5.09±0.12	5.29±0.11	5.20±0.08	4.33±0.13

**Methylmalonic Acid**	5.46±0.19	5.35±0.07	5.41±0.33	5.50±0.14	5.45±0.16	5.88±0.21
**N-Acetylmuramate**	4.69±0.25	4.52±0.16	4.49±0.15	4.53±0.17	4.46±0.35	3.30±0.44
**2-Oxoglutaric Acid**	4.39±0.43	4.20±0.14	4.21±0.37	4.34±0.22	4.41±0.14	3.99±0.17
**Propionic Acid**	4.22±0.32	4.06±0.11	4.13±0.41	4.29±0.25	4.15±0.24	4.65±0.27
**Fumaric Acid**	4.24±0.16	4.40±0.12	4.62±0.14	4.57±0.18	4.58±0.18	4.66±0.22
**Deoxyribose**	5.18±0.22	5.36±0.07	5.34±0.13	5.43±0.13	5.39±0.15	5.56±0.14
**Citric Acid**	5.80±0.10	5.99±0.16	5.89±0.09	5.94±0.04	5.96±0.05	5.97±0.08
**L-Threonic Acid**	6.09±0.30	6.29±0.11	6.07±0.19	6.19±0.19	6.09±0.29	6.42±0.16
**Fucose**	4.35±0.10	4.37±0.06	4.30±0.09	4.48±0.10	4.42±0.09	4.87±0.22
**Beta-Alanine**	6.55±0.35	6.89±0.05	6.91±0.12	7.02±0.05	6.89±0.14	6.51±0.18
**Ascorbic Acid**	3.67±0.47	4.07±0.71	4.36±0.88	4.30±0.82	4.12±1.01	3.09±0.04
**D-Glucuronolactone**	5.24±0.24	5.38±0.10	5.21±0.18	5.43±0.18	5.38±0.17	4.57±0.26
**Glyceric Acid**	5.01±0.17	5.26±0.17	5.34±0.24	5.34±0.17	5.17±0.40	5.17±0.20
**Cellobiose**	5.93±0.14	5.95±0.15	5.45±0.47	5.95±0.24	5.76±0.29	5.27±0.07
**Acetoacetic Acid**	4.96±0.05	4.86±0.08	4.75±0.05	4.84±0.10	4.76±0.10	4.80±0.05
**Sedoheptulose**	5.52±0.24	5.62±0.09	5.57±0.07	5.74±0.10	5.62±0.14	5.59±0.17
**Alpha-D-Glucose**	6.02±0.07	5.81±0.15	5.51±0.31	5.78±0.21	5.64±0.12	5.47±0.25
**L-Glutamine**	5.32±0.09	5.34±0.08	5.16±0.18	5.24±0.05	5.20±0.06	5.34±0.11

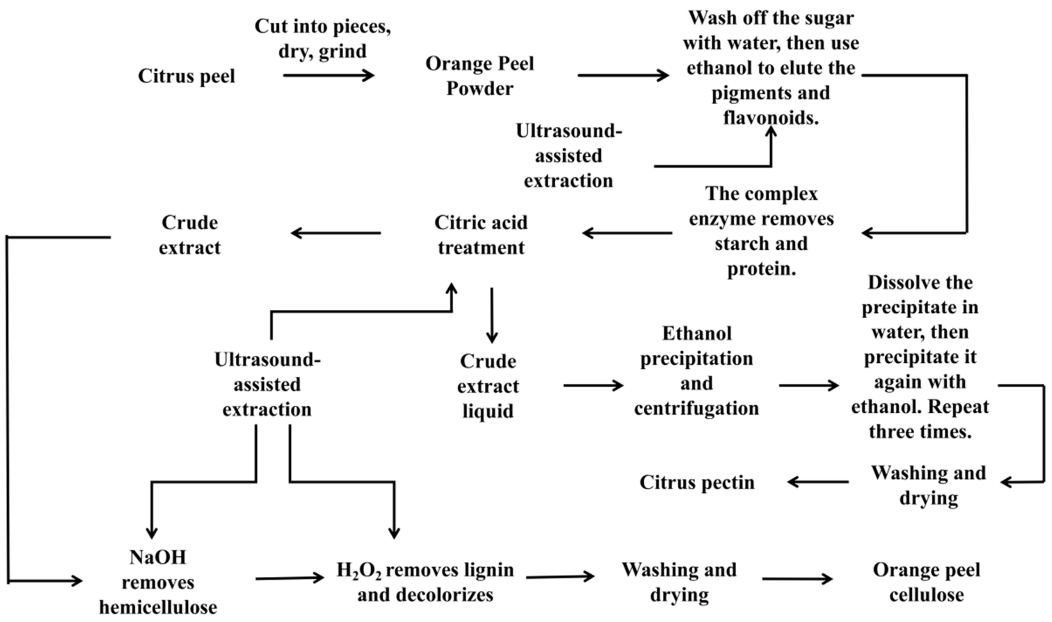

Supplement Figure S1. Process Flow Chart for CEL and PEC Preparation

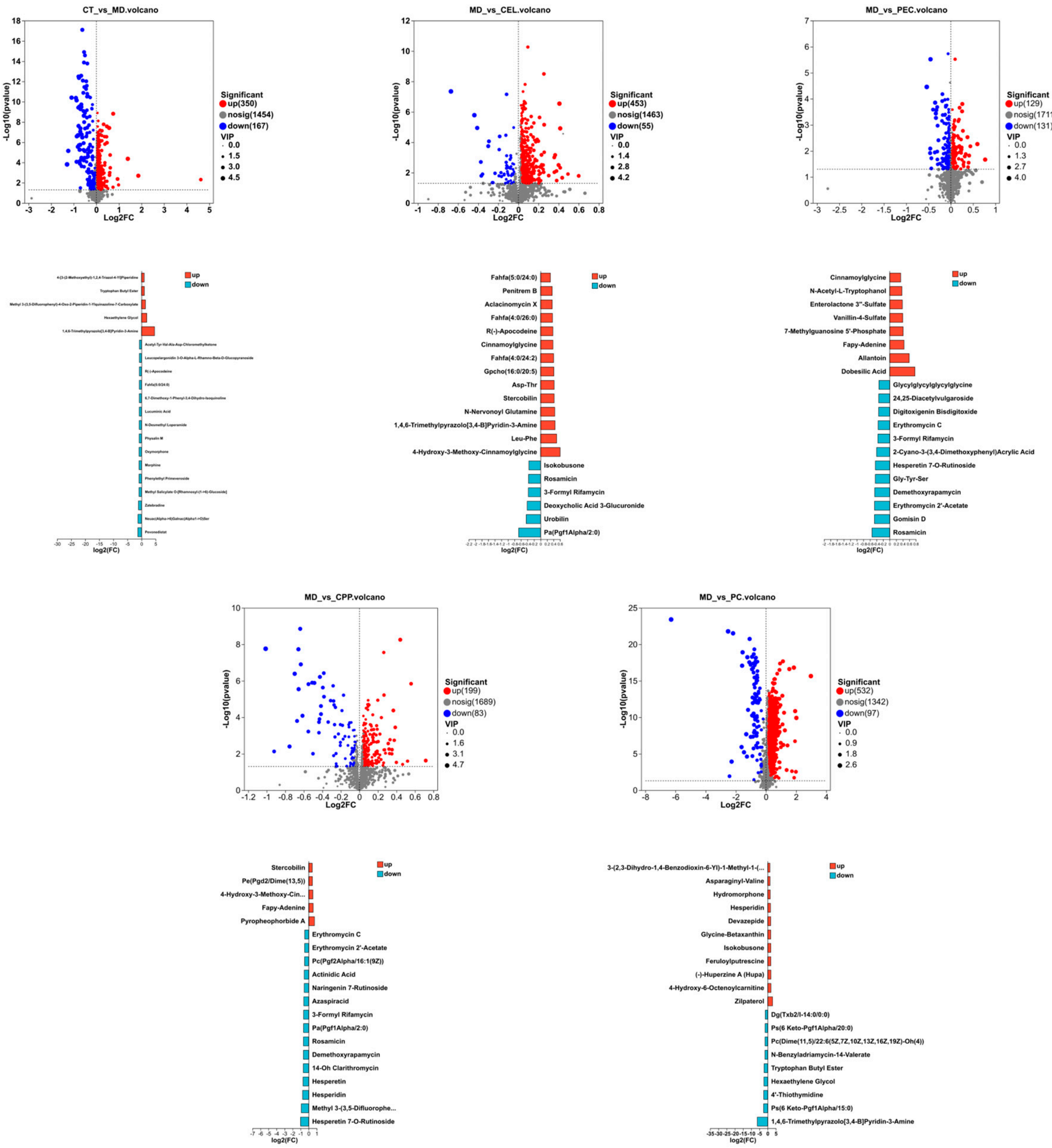

Supplement Figure S2. Volcano Plot and Differential Metabolite Statistics Chart

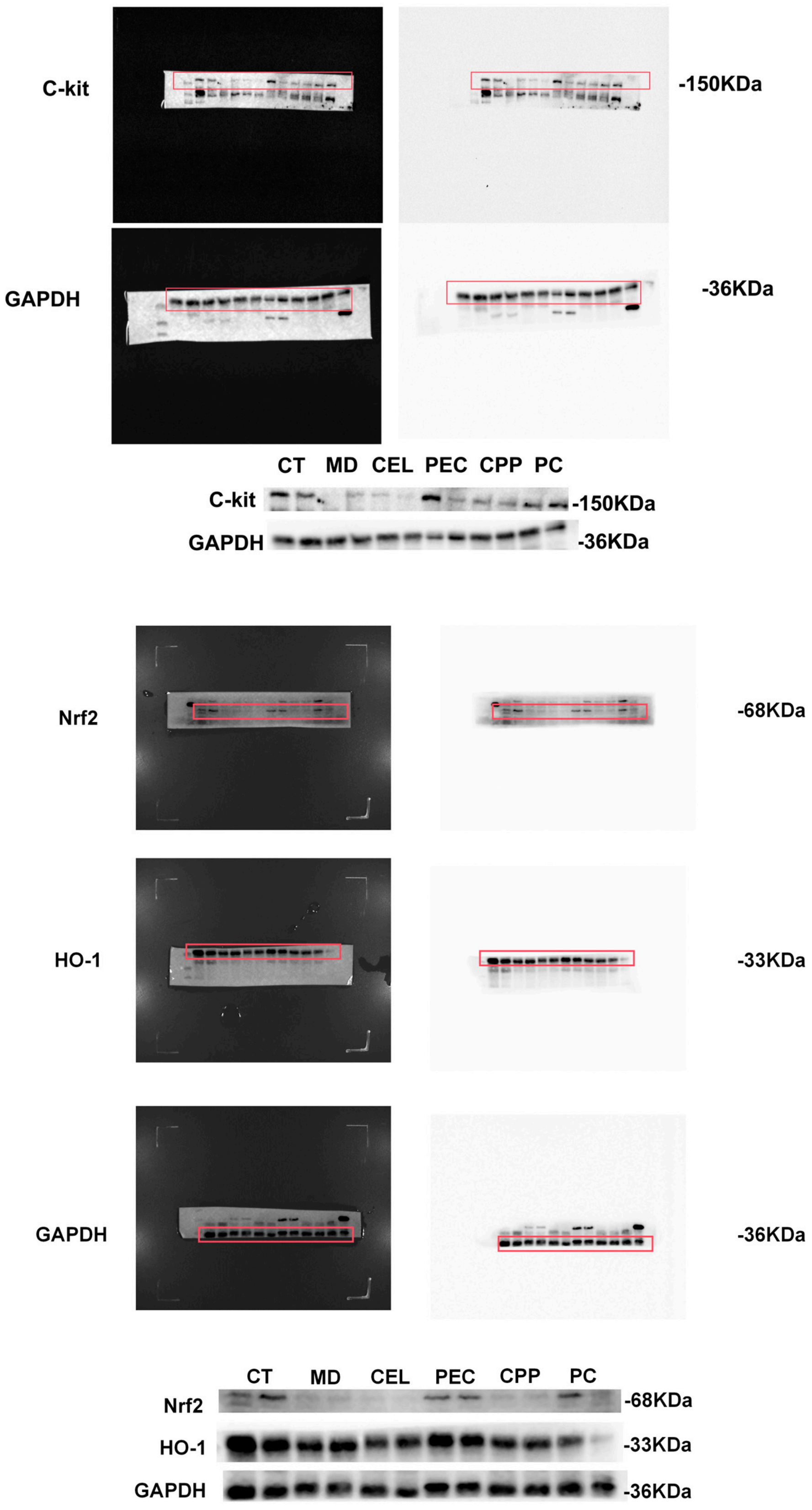

Supplement Figure S3. Western Blot Original Image
